# Screening for psychotherapy readiness with the University of Rhode Island Change Assessment Scale and the Readiness for Psychotherapy Index

**DOI:** 10.3389/fpsyg.2025.1530713

**Published:** 2025-08-12

**Authors:** Oliver Rumle Hovmand, Nina Reinholt, Anne Bryde Christensen, Bo Bach, Sidse Marie Arnfred

**Affiliations:** ^1^Psychiatry South, Region Zealand Mental Health Service, Vordingborg, Denmark; ^2^Department of Clinical Medicine, Faculty of Health, University of Copenhagen, Copenhagen, Denmark; ^3^Psychiatric Research Unit, Region Zealand Mental Health Services, Slagelse, Denmark; ^4^Center for Eating and Feeding Disorders Research, Mental Health Center Ballerup, Ballerup, Denmark; ^5^Department of Psychology, University of Copenhagen, Copenhagen, Denmark

**Keywords:** RPI, URICA, readiness, URICA scale, RPI scale, psychotherapy

## Abstract

**Background:**

It is important to assess patients’ level of readiness before starting a course of psychotherapy, but only a few validated instruments are currently available for that purpose.

**Methods:**

Patients waiting for psychotherapy were administered the Danish translations of the University of Rhode Island Change Assessment Scale (URICA) and the Readiness for Psychotherapy Index (RPI) prior to commencing psychotherapy. We conducted confirmatory factor analysis of the instruments’ previously suggested factor structure, as well as evaluating the instruments’ ability to identify early adherence to psychotherapy by receiver operating characteristic (ROC) curves and regression analysis.

**Results:**

One hundred and fifty-five patients with emotional, personality, and post-traumatic stress disorders were included. The instruments showed poor to average psychometric properties and poor predictive validity regarding early adherence to psychotherapy.

**Discussion:**

Findings indicated that the Danish URICA and RPI possess average psychometric properties and have limited validity for predicting early adherence to psychotherapy in psychiatric outpatients awaiting psychotherapy.

## Background

Research shows that many patients offered psychotherapeutic treatment are ambivalent toward it and may not be ready to participate (Engle and Arkowitz, 2006; Hunter et al., 2014; Oliveira et al., 2021). Data on cognitive group therapy from the Danish TRACT-RCT indicates high dropout rates in Danish outpatient mental health service (MHS) clinics—an average of 35.5% (*N =* 291) (Reinholt et al., 2021). This is somewhat consistent with international meta-analytic findings, which show dropout rates of similar magnitude (21.9%) (Swift et al., 2017).

These dropout rates may result in a substantial waste of human resources, and is particularly regrettable in settings offering group therapy, where dropouts may result in both direct economic costs due to wasted resources and indirect costs due to disturbance of the group dynamics (Wierzbicki and Pekarik, 1993; Barrett et al., 2008). Further, they may potentially indicate that people who need help and have been seeking it are not receiving it.

Factors that presently inform the clinical choice of psychotherapy as a relevant treatment modality include the presence of interpersonal problems (Barkham et al., 2021), the patient’s recognized need for help and capacity to relate (Barkham et al., 2021), patient preference (Williams et al., 2016) and psychological-mindedness (McCallum and Piper, 1997), and the patient’s motivation or readiness for change (Vogel et al., 2006). Relevant screening tools might help detect patients who are not ready for psychotherapy and may require other treatment efforts instead—i.e., motivational enhancement pre-therapy or other treatment modalities.

A patient’s motivation for change plays a crucial role in the outcome of psychotherapy (Vogel et al., 2006). The overall concept of motivation relates to the intentional aspect of change, such as the internal drive preceding the initiation of therapy, as well as the patient’s ongoing engagement throughout treatment (Prochaska and DiClemente, 2005), including the desire to change (Truant, 1999), willingness to discuss personal matters (Krause, 1967), level of distress (Schneider and Klauer, 2001), and preparedness to make reasonable sacrifices (Hacker, 1962).

Theoretically, the concept of readiness for change is described as a core component in the “stages of change” cycle of the so-called Transtheoretical Model (TTM) of behavioral change set forward by Prochaska and DiClemente (1984). In the “stages of change” cycle, patients are assumed to vary in their overall motivation to change, being on different levels ranging from having no intention for therapeutic change (“precontemplation”), to being ambivalent about change (“contemplation”), to having intentions and a commitment to change (“preparation”), to starting changes and actively working on them (“action”), to consolidating changes in order to prevent relapse (“maintenance”). According to the model, patients continuously move through the individual stages in a spiral pattern, and relapses are integral to the cycle. Cycling back to an earlier stage of change gives the patient the opportunity to apply different and possibly more effective strategies to make the changes they were not successful with the first time (Prochaska and Norcross, 2014).

Several measures have been used to evaluate motivation for change, of which the University of Rhode Island Change Assessment (URICA) is one of the most commonly employed (DiClemente and Hughes, 1990). The URICA is designed to measure readiness for change in regards to any behavior (e.g., behavioral change aimed at weight loss, substance abuse, etc.). It assesses this motivation dimensionally, with four dimensions derived from the TTM: Precontemplation, Contemplation, Action, and Maintenance. It is commonly calculated as a Readiness To Change (RTC) score, which is a composite score of a patient’s overall readiness derived from the individual subscales. In addition to this, a Committed Action (CA) measure has been proposed by Pantalon et al. (2002), based on the reflection that many of the items on the Contemplation subscale reflect ambivalence about change, and that endorsement of these items may reflect a decreased likelihood of taking action to change (Pantalon et al., 2002).

Aside from the URICA, a few instruments exist that are specifically designed to assess readiness for psychotherapy. One is the Counselling Readiness Scale (CRS), a subscale of the Adjective Check List (Gough, 1980) designed to identify persons who are open to change and who seem likely to profit from psychotherapy. Herein, respondents are asked to endorse adjectives which they believe accurately describe them. The CRS includes different scales for men and women with different numbers of items. Only limited information exists about the instrument’s reliability and validity, though studies conducted by its developers (Heilbrun, 1966; Heilbrun, 1978) found it helpful in predicting premature termination of therapy. Another example is the Stages of Change Readiness and Treatment Eagerness Scales (SOCRATES), which was developed as an alternative to the URICA for measuring readiness for change with regard to problem drinking (Miller and Tonigan, 1996). The SOCRATES includes 19 items which together make up three scales, ambivalence, recognition, and taking steps, which are hypothesized to represent continuously distributed motivational processes.

A third relevant instrument is the Readiness for Psychotherapy Index (RPI), developed by Ogrodniczuk et al. (2009) as a clinical tool to assess a patient’s readiness to enter into a psychotherapeutic intervention. Its creators amassed a pool of items from existing literature and clinical insight that were expected to reflect elements of readiness for psychotherapy. This resulted in a pool of 42 items that the authors believed to represent seven dimensions of readiness. From this pool, a final set of 20 items were selected and, through exploratory factor analysis, were found to be optimally described by a four-factor model. These factors encompass Disinterest, Perseverance, Openness, and Distress, and each one contains five items. An Overall Readiness score is calculated based on the four. High internal consistency was reported by the developers (Ogrodniczuk et al., 2009).

Trials have consistently found patients’ readiness to change to be an essential factor in predicting and moderating psychotherapy outcomes (Ogrodniczuk et al., 2009). Thus, a valid measure of this construct is critical to understanding its potential impact in specific contexts or populations, such as in the treatment of mood and personality disorders, eating disorders, and addiction. Readiness to change is particularly relevant in these populations since treatment consists primarily of psychotherapeutic interventions, which require much effort and persistence, and since there is an inherent low motivation or ambivalence toward change in these disorders (i.e., lack of energy and initiative in depression, ambivalence toward behavioral change in eating disorders and addiction).

The URICA has mainly been applied in research on substance (Abellanas and McLellan, 1993; Belding et al., 1995; Carney and Kivlahan, 1995; Belding et al., 1997; el-Bassel et al., 1998; Field et al., 2009) and alcohol use disorders (DiClemente and Hughes, 1990; Field et al., 2009; Willoughby and Edens, 1996; DiClemente et al., 1999; Edens and Willoughby, 1999; Carbonari and DiClemente, 2000; Edens and Willoughby, 2000; Hoyer et al., 2003), but has also been used in studies on other psychiatric disorders such as anxiety disorders (Dozois et al., 2004) and bulimia nervosa (Treasure et al., 1999). It has additionally been applied to patients with somatic pain (Kerns and Rosenberg, 2000), criminal offenders (Polaschek et al., 2010), tobacco smokers (DiClemente et al., 1991), and individuals wishing to lose weight (Ingledew et al., 1998).

Krebs et al. (2018) conducted a meta-analysis of the association between measures of readiness and psychotherapy outcomes. They included 76 studies, in which the most frequent readiness measures were the URICA (*n* = 46) and the SOCRATES (*n* = 10). Thirty-six studies provided data on the association between readiness and adherence to treatment/premature dropout. Among these reports, the authors found a medium mean effect size (d = 0.36) (95% CI = 0.26–0.47) (Krebs et al., 2018).

Two studies have investigated the use of RPI in populations with emotional and substance-related disorders awaiting psychotherapy (Ogrodniczuk et al., 2009; Kealy et al., 2017). Another has investigated the relationship between psychosis spectrum symptoms and psychotherapy readiness among inpatient adolescents in the mental health service (Thompson et al., 2021). None of these examined the instrument’s external validity regarding adherence to subsequent psychotherapy.

However, the utility of these two instruments for assessing readiness for psychotherapy engagement or adherence among patients with emotional disorders has only been analyzed in one study (Dozois et al., 2004). Further, they have yet to undergo psychometric validation in Danish. Hence, the objectives of the present study are to examine the psychometric properties of a public Danish translation of the URICA and our own Danish translation of the RPI and evaluate their predictive validity through their ability to predict early adherence to group psychotherapy.

We hypothesize that the Danish URICA and RPI both have a four-factor structure corresponding to the one suggested for the American (Dozois et al., 2004) and Canadian English originals (Ogrodniczuk et al., 2009). Additionally, we expect them to be able to adequately discriminate between patients who are ready or not ready for psychotherapy, as judged by their early adherence to at least three of their first four planned sessions of psychotherapy as a proxy measure of psychotherapy engagement.

## Methods

### Setting

**Figure 1:**
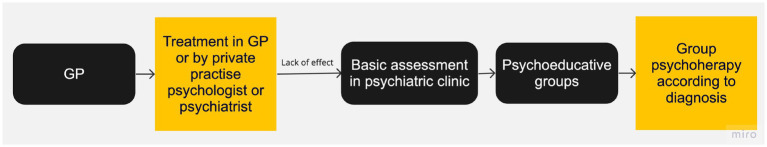
Patient flow in and through the clinic.

Patients were recruited in outpatient secondary care clinics run by Region Zealand Mental Health Services (MHS). Patients can be referred to these clinics if they have failed to respond to one line of treatment in the primary care sector. In the MHS clinics, patients undergo diagnostic assessment at the intake interview and receive therapy in a group according to diagnosis. While the patients are waiting for specialized treatment, they are offered psychoeducational group sessions (see Figure 1 for a representation of patient flow in the clinic). The first author attended these sessions between March 2022 and January 2023 and recruited participants for the present study. A subsample of patients were included through participation in a randomized controlled trial in the same clinics, also during the waiting period before group psychotherapy (Hovmand et al., 2023).

### Participants

The participants had primary diagnoses of emotional disorders, post-traumatic stress disorder (PTSD), or personality disorders. Patients were only eligible for treatment in the MHS clinics if they had been subjected and not responded sufficiently to primary care treatment. Thus, this group might be characterized, at least partially, as a treatment resistant sample.

### Procedure

After informed, written consent, the patients were administered the self-report scales. Subsequently, information regarding primary diagnosis, co-occurring disorders, and adherence to psychoeducation and psychotherapy were drawn from the patients’ electronic health records. If a reason was given for non-adherence or for declining the offer to receive therapy, this was recorded.

Data regarding adherence to psychotherapy were collected until the end of April 2023. Patients who dropped out prior to being offered therapy were coded as non-completers. Patients who were still waiting for onset of psychotherapy as of April 2023 were not included in the predictive validation analysis. This is reflected in Table 1 “Completers” refers to the subsample of patients who was offered psychotherapy during the period of data collection, and which attended three or more sessions out of the first four sessions. The “non-completers” do in the same table, refer to the patients of the same population, which did not.

**Table 1:** URICA scores on patients who attended or did not attend their first four sessions of psychotherapy in a group.

*n* (%)	*N*	Precontemplation	Contemplation	Action	Maintenance	CA	RFC
Total sample	155	13.3 (3.8)	35.36 (2.9)	32.6 (3.9)	31.1 (3.9)	−2.7 (3.2)	85.9 (10.1)
Completers	70	12.6 (3.7)	35.64 (2.97)	33.0 (3.6)	31.1 (3.7)	−2.64 (2.95)	87.1 (9.6)
Non-completers	16	13.7 (3.1)	36.0 (3.0)	32.3 (4.7)	31.3 (4.4)	−3.7 (3.1)	85.8 (11.6)

Completers and non-completers refers to those who did and did not attend three or more of their first four therapy sessions. Standard deviations are in parenthesis. Completers refers to the subsample of patients who was offered psychotherapy during the period of data collection, and which attended three or more sessions out of the first four sessions. The non-completers refers to the patients of the same population, which did not.

### Ethical considerations

The study was in accordance with local regulations registered with the Danish Data Protection Agency Region Zealand (REG-170-2021). The survey study did not, as per local guidelines and regulations, need approval by the Region Zealand Ethics Committee. However, the randomized clinical trial from which a subsample of participants’ data were drawn was approved by the Region Zealand Ethics Committee (Registration number: SJ-924) and registered with the Region Zealand Data Protection Agency (Registration number: REG-050–2021). Informed consent was collected from participants in order to join the study.

### Instruments

#### University of Rhode Island change assessment scale (URICA)

The URICA is a 32-item self-report measure designed to quantify the patient’s motivation for change. It includes the following four subscales: *Precontemplation*, *Contemplation*, *Action*, and *Maintenance.* Each of the subscales includes eight items, and these are rated on a Likert-type scale ranging from 1 to 5, with higher scores indicating greater endorsement of particular behaviors or attitudes (1 = Strongly disagree; 2 = Disagree; 3 = Undecided; 4 = Agree; 5 = Strongly agree) (DiClemente and Hughes, 1990).

The score for each subscale is calculated by adding the score for each item. In addition, two further composite scores can be calculated from this information. The Readiness To Change (RTC) composite score is obtained by subtracting the Precontemplation and Contemplation scores from the sum of the Action and Maintenance scores. The Committed Action (CA) score is the Contemplation score subtracted from the Action score (Pantalon et al., 2002). Total discrete stage scores range from 8 to 40 for each subscale, whereas RTC scores range from −16 to 112. For the CA, scores range from −32 to 32.

#### Readiness for psychotherapy index (RPI)

The RPI is a 20-item self-report measure that was developed especially to assess readiness for psychotherapy. The items load onto the following four factors: disinterest (items relating to lack of interest and lack of belief that therapy is needed); Perseverance (items relating to the patient’s willingness to endure and work in therapy); Openness (items relating to the patient’s willingness to discuss personal matters with the therapist and accept discomfort in doing so); and Distress (items relating to the patient’s level of discomfort and feeling of urgency) (Ogrodniczuk et al., 2009). Each factor includes five items, which are rated on a 5-point Likert scale ranging from strongly disagree (1) to strongly agree (5). An Overall Readiness score is calculated by subtracting the Disinterest subscale’s score from the others’ total. The total discrete Overall Readiness score ranges from −10 to 70.

#### Level of personality functioning scale—brief form 2.0 (LPFS-BF)

Since personality functioning was considered an important modifier of adherence to psychotherapy, independent of level of readiness, we also collected data using the LPFS-BF 2.0. This is a 12-item self-report measure designed to assess impairment in self and interpersonal functioning (Weekers et al., 2019). The 12 items are designed to capture 12 capacities that, all together, portray the global level of personality functioning corresponding to the criterion A of the Alternative Model for Personality Disorders (AMPD) in DSM-5 Section III (Krueger et al., 2012; Association, American Psychiatric, 2013), as well as partially to the ICD-11 classification of personality disorder severity (Bach et al., 2017; WHO, 2022). Respondents rate the items on a 4-point Likert scale (0 = Very false or often false; 1 = Sometimes or somewhat false; 2 = Sometimes or somewhat true; 3 = Very true or often true) (Bach and Hutsebaut, 2018). We used a Danish-validated translation of the instrument (Weekers et al., 2023).

### Translations

For the URICA, we utilized a Danish translation clinically applied in Denmark.

For the RPI, we secured permission from Ogrodniczuk et al. (2009) to translate and validate the instrument in a population of Danish patients awaiting psychotherapy. We then translated it according to established guidelines (Hambleton, 2001). Briefly, an expert panel translated the instrument using the backward–forward method. After the initial translation, we collected user feedback on this version from patients with anxiety disorders. The expert panel met again, discussed user input, and made a new version, which was then translated to English by a bilingual medical professional who had worked in the Danish MHS for 4 years. The back translation was presented to Ogrodniczuk, who commented on it, and a final Danish edition was made and translated to Canadian English. This last version was approved by Ogrodniczuk in 2021. The result of the translation is presented in Supplementary material.

### Data processing and statistics

We undertook all data processing and analyses using R 4.3.0 (Already Tomorrow) and RStudio 2022.07.2 + 576 (Team R Studio, 2019), including the psych 2.1.9 (Revelle, 2017) and lavaan 0.6–9 (Rosseel, 2012) R packages. R scripts are available from the first author upon request.

First, we calculated descriptive statistics, conducting unpaired sample *t*-tests to examine if there was a difference between scores for completers and non-completers.

Secondly, we conducted confirmatory factor analyses of the Danish URICA and RPI to estimate their fit with the four-factor model suggested by previous factor analyses. We compared the original suggested four factor structure, to a unidimensional single-factor model where all items loaded on one factor. To do so, we calculated the comparative fit index (CFI), the Tucker-Lewis index (TLI), the root means square error of approximation (RMSEA), the standardized root mean square residual (SRMR), and the degrees of freedom (df). The consensus is that a larger RMSEA and smaller CFI and TLI values indicate a worse fit (Xia and Yang, 2019). More specifically, we utilized the criteria set forth by Hu and Bentler, which suggest that an RMSEA smaller than 0.06, an SRMR close to 0.08, and a CFI and TLI larger than 0.95 indicate relatively good model–data fit in general (Hu, 2009).

For the URICA specifically, we also evaluated whether omitting the items with the lowest loading from each factor, would improve model fit. We therefore examined revised model 1 where the items with the poorest loading in each factor was dropped, and revised model 2 where the two items with the poorest loading in each factor was dropped.

Third, we assessed the reliability of the Danish URICA and RPI by calculating Cronbach’s alpha (*α*), MacDonald’s hierarchical (ωh), and the total omega (ωtotal) for both the complete scales and each of the individual factors. Cronbach’s alpha and omega above 0.70 were considered satisfactory (Bland and Altman, 1997; Hair et al., 2016).

Lastly, we evaluated the predictive validity of the scales by performing a receiver operating characteristic (ROC) analysis to determine the area under the curve (AUC) and their specificity and sensitivity regarding adherence to psychotherapy (attended three or more of the first four sessions, yes/no). We coded those who did not attend as non-completers, regardless of reason, and utilized the following criteria set forth by Metz (1978) to interpret the results: 0.5–0.6 (failed), 0.6–0.7 (worthless), 0.7–0.8 (poor), 0.8–0.9 (good), > 0.9 (excellent).

To test whether the URICA RFC and the RPI Overall Readiness scores significantly predicted adherence to psychotherapy (percentage of no-shows in the first four sessions), we conducted a logistic regression with and without LPFS-BF as a co-factor and with and without time spent waiting as a cofactor. We did so both for the original instrument but also for the revised model 2.

We also calculated the Pearson’s correlation between the RPI and its subscales and the URICA in order to examine the convergent validity. We utilized the following criteria set forth by Cohen (1988) to interpret the results: 0.1 (small); 0.3 (medium) and 0.5 (large).

## Results

Data collection took place between December 2021 and April 2023. A total of 155 patients gave informed consent and were included in the study. One hundred and five were recruited from the psychoeducational groups and 40 enrolled in the RCT.

The sample had a mean age of 31.1 (SD = 10.8) years. Most (79.3%) were female and were on sick leave (43.9%) or unemployed (14.8%). Most were awaiting treatment for avoidant personality disorder (32.9%) or borderline personality disorder (30.2%). Further, the 86 patients who were offered therapy during the study waited for a mean 10.0 (SD = 5.9) months from the time of their first appointment in the clinic to their first psychotherapy session. See Table 2 for further demographic and diagnostic details of the included sample.

**Table 2:** Sample characteristics.

Total (*n* = 155)	
Female	123 (79.35%)
Master’s or bachelor’s degree	25 (16.13%)
Employment	
Full time/part time/student	64 (41.29%)
Sick leave	68 (43.87%)
Unemployed	23 (14.84%)
Principle diagnosis	
Unipolar depression	18 (11.61%)
Avoidant personality disorder	51 (32.90%)
Borderline personality disorder	47 (30.25%)
Post-traumatic stress disorder	14 (9.03%)
OCD	5 (3.22%)
Anxiety disorders	17 (10.97%)

Data are presented as *n* (%).

For eight patients (5.2%), there was a reason documented in the electronic health records for why the patient could not attend therapy. One had a child with a health condition, four had a somatic health condition of their own, and three could not attend due to lack of time.

### URICA

The mean URICA RTC score across diagnostic groups was 85.9 (SD = 10.1), corresponding to 76.9% of the maximum composite scale score. The mean URICA RTC score for the 70 patients who were offered therapy and attended ≥ 3 of the first four sessions (“completers”) was correspondently 87.1 (SD = 9.6), and the score for the 16 patients who did not (“non-completers”) was correspondently 85.9 (SD = 11.6). See Table 1 for summary statistics regarding the scores on each URICA subscale for the entire sample, completers, and non-completers. There was no difference between the mean scores of the completers and non-completers (t = 0.39696, df = 19.972, *p* = 0.7).

### Factor structure of the Danish URICA

Table 3 presents the fit statistics for the evaluated factor structures. The original four-factor model had only a poor to reasonable fit.

**Table 3:** Fit statistics for two different possible factor structures of the Danish URICA.

Model	CFI	TLI	RMSEA	SRMR	Chi^2^	Df
Single-factor model	0.871	0.862	0.112	0.129	1364.256	494
Four-factor model	0.933	0.927	0.082	0.110	927.299	458
Revised four-factor model 1*	0.955	0.951	0.072	0.103	616.052	344
Revised four-factor model 2 **	0.964	0.960	0.071	0.100	434.311	276

*N* = 155. CFI, comparative fit index; TLI, Tucker-Lewis index; RMSEA, root mean square error of approximation; SRMR, standardized root mean square residual; Df, degrees of freedom. *Items 23; 19; 10; 28 was dropped. **Items 23; 31; 19; 12; 10; 20; 28; 16 was dropped.

As a *post-hoc* analysis, we investigated whether fit could be improved by systematically omitting items with the most unsatisfactory loadings. In a first step, we omitted a total of four items corresponding to one item from each of the four factors (items 23, 19, 10, and 28). In a second step, we omitted a total of eight items, corresponding to the two most poor-performing items from each of the four factors (items 23, 31, 19, 12, 10, 20, 28, and 16).

As shown in Table 3, these tentative modifications improved model fit.

**Figure 2:**
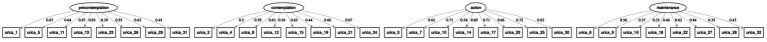
Factor loadings of the four-factor model of the Danish URICA.

Figure 2 presents factor loading patterns for the original four-factor model.

### Reliability and internal validity of the Danish URICA

Internal consistencies were found to be good for most of the individual subscales. See Table 4 for values for each individual subscale.

**Table 4:** RPI scores on patients who attended or not-attended the first four sessions of psychotherapy in a group.

*n* (%)	*N*	Disinterest	Perseverance	Openness	Distress
Total sample	159	8.4 (2.3)	21.1 (2.1)	18.3 (4.0)	16.6 (2.2)
Completers	72	8.1 (2.3)	21.3 (2.2)	18.3 (3.5)	16.5 (2.2)
Non-completers	16	7.8 (2.0)	20.8 (2.2)	18.2 (4.0)	16.5 (2.2)

Completers and non-completers refers to those who did and did not attend three or more of their first four therapy sessions. Standard deviations are in parenthesis.

**Figure 3:**
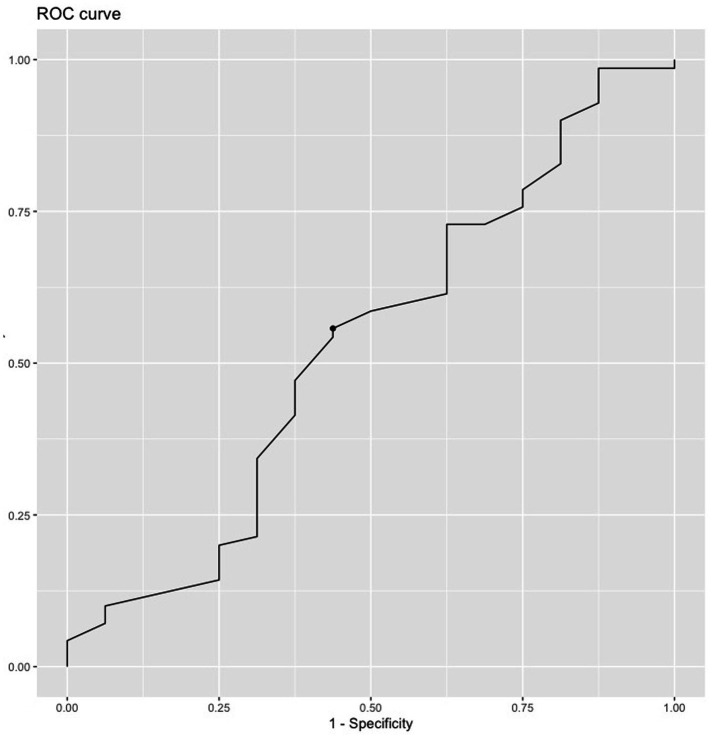
ROC curve of URICA testing for sensitivity to adherence to psychotherapy (to ≥ 3 of the first four psychotherapy sessions offered). Dot represents optimal cut-off point (*N* = 86).

ROC curve testing for sensitivity to early adherence to psychotherapy (attendance to ≥ 3 of the first four sessions of therapy for the selected sample of patients) (*N =* 86) is presented in Figure 3. Sixteen patients did not attend ≥ 3 of the first four sessions of therapy. The optimal cut-off point on the URICA Readiness To Change score across diagnostic groups was 87. It yielded a sensitivity of 56% and a specificity of 56% with failed accuracy, as indicated by an AUC = 0.53.

The optimal cut-off point for the URICA total score was 107. This yielded a sensitivity of 86% and a specificity of 17% with failed accuracy, as indicated by an AUC = 0.47.

For the Revised four factor model 1, the optimal cut-off point for the URICA total score was 94. This yielded a sensitivity of 86% and a specificity of 17% with failed accuracy, as indicated by an AUC = 0.48.

The Revised four factor model 2 likewise had an optimal cut-off point for the URICA total score of 84. This yielded a sensitivity of 81% and a specificity of 19% with failed accuracy, as indicated by an AUC = 0.48.

The URICA RTC score did not predict early adherence [R^2^ = 0.0007184, *F*_(1, 84)_ = 0.06038, *p* = 0.8], and neither did linear regression analysis for any other URICA subscales. We also evaluated the URICA RFC in combination with LPFS-BF and the LPFS-BF in combination with each URICA subscale, but these did not predict early adherence either. Lastly, we evaluated the URICA RFC and each of the URICA subscales in combination with time spent waiting, but this also failed to predict early adherence.

We also evaluated these for the revised four factor model 1 and the revised four factor model 2, but these did as well not predict early adherence.

### RPI

The RPI Overall Readiness score for the entire sample was 47.5 (SD = 6.9). The RPI Overall Readiness score for the 72 patients who were offered therapy and attended ≥ 3 of the first four sessions (“completers”) was correspondently 48.0 (SD = 6.6), and the score for the 16 patients who did not (“non-completers”) was correspondently 47.7 (SD = 6.8). There were no differences in the mean scores of RPI Overall Readiness between the completers and non-completers (*t* = 0.1415, df = 21.931, *p* = 0.88). See Table 4 for descriptive statistics regarding scores on the RPI for the entire sample, completers, and non-completers.

### Factor structure of the Danish RPI

**Figure 4:**
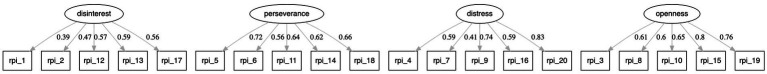
Factor loadings of the four-factor model of the Danish RPI.

Table 5 presents the fit statistics for the factor structures. The model is graphically presented in Figure 4. The four-factor model had the best fit of the two models evaluated. Figure 3 presents factor loading patterns for this model.

**Table 5:** Fit statistics for two different possible factor structures of the Danish URICA.

Model	CFI	TLI	RMSEA [90% CI]	SRMR	Chi^2^	Df	Chi^2^/Df
Single-factor model	0.871	0.862	0.112 [0.105; 0.119]	0.129	1364.256	494	2.76
Four-factor model	0.933	0.927	0.082 [0.074; 0.089]	0.110	927.299	458	2.05
Revised Four-factor model 1*	0.955	0.951	0.072 [0.061; 0.079]	0.103	606.019	344	1.76
Revised Four-factor model 2 **	0.964	0.960	0.071 [0.060; 0.081]	0.100	434.311	276	1.57

*N* = 155. CFI, comparative fit index; TLI, Tucker-Lewis index; RMSEA, root mean square error of approximation; SRMR, standardized root mean square residual; Df, degrees of freedom. *Items 23; 19; 10; 28 was dropped. **Items 23; 31; 19; 12; 10; 20; 28; 16 was dropped.

### Reliability and validity of the Danish RPI

Internal consistency was found to be satisfactory for the Openness subscale, but not for the remaining subscales. See Table 4 for values for each individual subscale.

### Convergent validity

The RPI Overall Readiness had a medium-to-large correlation with the URICA RFC score (r = 0.42, 95% CI [28, 0.54], *p* ≤ 0.001). The Distress subscale of the RPI had a medium correlation with the LPFS-BF (r = 0.38 95% CI [24, 0.51], *p* ≤ 0.001).

### Predictive validity

**Figure 5:**
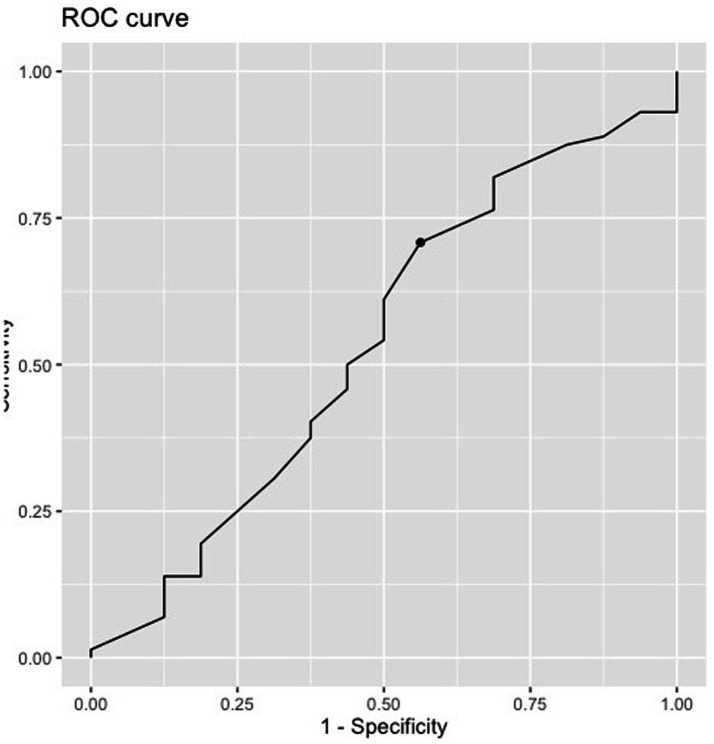
ROC curve of RPI testing for sensitivity to adherence to psychotherapy (adherence to ≥ 3 of the first four psychotherapy sessions offered). Dot represents optimal cut-off point (*N* = 86).

ROC curves for the eligible sample of patients (*N =* 88) are presented in Figure 5. Sixteen patients did not attend ≥ 3 of their first four sessions of therapy. The optimal cut-off point for RPI Overall Readiness score across diagnostic groups was found to be 45; it yielded a sensitivity of 71% and a specificity of 44% with failed accuracy, as indicated by an AUC = 0.66.

RPI Overall Readiness did not predict adherence to therapy [R^2^ = 0.001651, *F*_(1, 86)_ = 0.001651, *p* = 0.7], nor did any of the subscales. We also evaluated RPI Overall Readiness in combination with LPFS-BF and the LPFS-BF in combination with each RPI subscale, but these did not predict early adherence either. We finally evaluated RPI Overall Readiness and each of the RPI subscales in combination with time spent waiting, but this likewise failed to predict early adherence.

Adding LPFS-BF to the prediction model did not change the results [R^2^ = 0.02003, *F*_(2, 85)_ = 0.8688, *p* = 0.42]. This was also the case for the LPFS-BF in combination with any RPI subscale.

## Discussion

The present study examined the psychometric characteristics of the Danish URICA and RPI in a clinical sample of patients with PTSD, mood, and personality disorders who were awaiting therapy. To the best of our knowledge, it is the first time the RPI has been investigated as a possible screening instrument. Our sample was heterogeneous regarding diagnosis, but all were patients in the publicly funded Danish Mental Health Services and were, therefore, all patients with complex psychopathology who had not sufficiently responded to previous attempts at treatment. The sample was highly motivated overall and had URICA scores of comparable magnitude on all subscales to populations with anxiety (Dozois et al., 2004), substance use (Pantalon et al., 2002), and bulimia nervosa (Treasure et al., 1999). Of previous research, only Dozois et al. (2004) study on patients with anxiety explored predictive validity regarding adherence to therapy. However, this study simply noted that patients completed the URICA before beginning therapy, not how long beforehand, and no information was given on the duration between completion of the URICA and the beginning of therapy.

We found only a poor-to-reasonable fit to the four-factor model which had previously been suggested for the URICA. This was, however, the best fit of the two different models evaluated. This reasonable fit is consistent with other studies on the URICA, which have likewise investigated the factor structure of the entire instrument with CFA (Pantalon et al., 2002; Field et al., 2009).

Similarly, we carried out CFA for the RPI and found a poor to reasonable fit to the four-factor model which had previously been suggested for this instrument. We are unaware of any other studies that have reported CFA fit measures for the RPI, as Ogrodniczuk et al. (2009) did not. This could suggest that the instruments do not, in fact, assess four distinctly different dimensions as has been theoretically suggested.

Neither the URICA, the RPI nor any of their subscales or composite measures predicted early adherence to therapy in our study. Nor did the Revised four factor model 1 or 2. This contrasts with findings by Dozois et al. (2004), who examined the English URICA’s ability to predict retention to cognitive behavior therapy in a sample of 81 patients recruited from a teaching hospital in Canada. They showed that the Action subscale predicted retention to therapy [*F*_(4, 76)_ = 2.66, *p* < 0.05] (Dozois et al., 2004). We are not aware of any studies that have examined the predictive power of RPI.

Our results suggest that there is no or insignificant association between readiness for therapy and early adherence to group psychotherapy for this patient group. If that is the case, other factors (e.g., demands from the social system that dictates that patients should seek and attend treatment to receive welfare benefits; relationships with other members of the therapeutic group; patients not wanting to lose access to their contact person in the clinic) could explain why some patients attend group psychotherapy despite low readiness. Some patients may also decline the offer to start group psychotherapy due to other obligations which do not allow them to attend in the daytime (e.g., employment or schooling conflicts; having to move to another city to start schooling; living with a somatic health condition). Hence, patients in full-time occupation may not receive an appropriate/feasible treatment offer with current clinical practice, which is designed for individuals who can attend therapy in the daytime and has little to no flexibility regarding time scheduling. The findings could thus signal that some patients should have been offered psychotherapy in the evenings, or flexible individual psychotherapy. Due to the naturalistic design of the study, patients were not systematically asked for the reason why they did not attend therapy. Future research should systematically investigate and record patients’ reasons for non-adherence or for declining therapy.

However, it is also possible that the lack of predictive validity reflects methodological shortcomings in the present study, which could have several explanations:

1. The URICA and RPI were administered to patients at the psychoeducational groups. For patients to reach these groups, they had already needed to take several steps (see Figure 1). As such, all the patients included in the sample had, at this point, shown a significant amount of persistence in their pursuit of treatment and had, on many occasions, had the chance to drop out of it. This could explain the homogeneous high readiness observed in the current sample, and the homogeneity of readiness in the sample plus the low number of no-shows could be the reason for the scales’ inability to predict adherence. In addition to this, only a limited number of the included patients were offered therapy within the duration of the study, and of these, only a few dropped out. Future studies should administer the instruments to patients at their first appointment in the clinic or at the start of therapy. Future research could also be conducted in a primary care setting, administering the instruments to patients who are referred to private practice psychologists from general practitioners, and investigate any association with adherence to private practice psychotherapy. On a related note, it is possible that patients’ motivation fluctuated over time, and that participants were either less or more motivated to change at the point in their treatment journey where the instruments were administered as compared to later. Administration of the instruments on several occasions could investigate whether this is the case. Further, the Transtheoretical Model (TTM) of behavioral change has received substantial criticism for not addressing the fact that a person may be in different stages of change at the same time for one single type of behavior (e.g., in the action stage regarding adherence to therapy, but in the contemplation stage regarding therapy homework). Two individuals may therefore be placed in the same stage despite having different perspectives regarding the behavior (e.g., in precontemplation, one patient may know that she is inactive but not be interested in changing her course of behavior, while another may be entirely unaware that he is inactive and needs to change). These two kinds of precontemplators might score the same on the URICA but still be entirely different. Another issue is that the TTM attempts to define stages with sequential transitions between them, but this phenomenon might be a continuum of change (e.g., a patient might be contemplating change but simultaneously reading about therapy online, i.e., taking small action steps). Such a patient’s stage on the TTM would be hard to define (Sussman et al., 2022). It might therefore be the case that the TTM and, in turn, the URICA lacks precision and does not capture the constructs necessary to assess readiness for behavioral change in a way that is relevant for psychotherapy.
2. It is also possible that early adherence as a marker of overall adherence and the cut-off of ≥ 3 were poor measures of overall adherence to therapy. Comparably, Dozois et al. (2004) defined non-completers as those who missed more than two out of eight total sessions without notification of absence and did not complete post-group evaluation. This threshold is looser than the one applied in the present study, as we did not allow for notification of absence and strictly counted attendance to the first four sessions of therapy. It is possible that our methods defined some patients as non-completers who would later resume attending the group. However, overall we find our outcome to be a relevant substitute for adherence to therapy, similar to the one utilized by Dozois et al. (2004).
3. Attendance alone might not necessarily provide information on whether a patient puts effort into therapy (e.g., participates in discussion, completes their therapeutic homework), or if they simply attend and sit in silence. Future studies could administer outcomes relating to involvement in therapy (e.g., completion of homework, therapist evaluation of patient’s involvement) and investigate a possible association between readiness and such outcomes.
4. Most patients waited for a considerable amount of time before being offered therapy. As a result, there was also a considerable period between the administration of the instruments and the beginning of therapy. Future research should be conducted in a setting where treatment can be provided to referred patients within a reasonable amount of time.
5. The URICA was originally designed for use on patients with alcohol addiction. It might therefore assess readiness for change in a way that is not relevant for patients with emotional disorders and personality disorders. This could be the reason for lack of association with early adherence to psychotherapy.

Next, we examined the internal consistency of the Danish URICA and RPI. The internal consistency of the action subscale was satisfactory, while the remaining subscales were found to be either borderline satisfactory or unsatisfactory. These measures of internal consistency were similar to early research on the original URICA (DiClemente and Hughes, 1990) (alpha for the individual subscales: Precontemplation = 0.69; Contemplation = 0.75; Action = 0.82; Maintenance = 0.80), and in patients with anxiety (alpha for the individual subscales: Precontemplation = 0.73; Contemplation = 0.79; Action = 0.90; Maintenance = 0.81) (Dozois et al., 2004) and cocaine use (alpha for the individual subscales: Precontemplation = 0.75; Contemplation = 0.79; Action = 0.83; Maintenance = 0.78) (Pantalon et al., 2002). However, they were moderately poorer than in research utilizing the original URICA in patients with other substance use (alpha between 0.80 and 0.84) (Carney and Kivlahan, 1995). We are unaware of any studies that have published an omega value for the URICA subscales. The poorer alpha values could possibly be explained by the fact that the original URICA was explicitly developed to assess motivation for change among alcohol and substance users, and such users might understand the individual items differently than individuals with anxiety and other psychiatric disorders.

Internal validity of the Danish RPI was broadly comparable to the alpha values in Ogrodniczuk et al. (2009) for subscale Openness (0.77 vs. 0.79), but was lower for subscales Disinterest (0.46 vs. 0.87), Perseverance (0.66 vs. 0.80), and Distress (0.63 vs. 0.71). Ogrodniczuk et al. (2009) did not publish omega values in their paper (Ogrodniczuk et al., 2009). These findings might suggest that the items in the Danish RPI related to disinterest, perseverance and distress are understood differently by Danish patients as compared to the original sample.

Limitations to the present trial include the fact that its results may generalize only to the included diagnoses and outpatient mental health services. In addition, the composition of the sample limits the generalizability of the results to males and to people older than 40 years. A limitation of the present study is the limited sample size, and especially the limited subsection of patients which did actually proceed to psychotherapy during the study period. Due to the limited sample size, we did not explore the possible impact of diagnosis or motivation on readiness for change. The study’s primary strength is its public mental health setting and inclusion criteria similar to those used in clinics, which adds to the external validity of the findings.

In conclusion, the predictive validity of the URICA and RPI are limited regarding early adherence to group psychotherapy in the Danish Mental Health Services. Our results suggest that they may not be useful as screening instruments; however, further study is needed. Future research in this area should administer the URICA and RPI upon intake to the clinic or at the start of therapy, or upon referral to the clinic from a general practitioner. Future research could also explore any possible influence of diagnosis and motivation on patients’ readiness for change.

## Data Availability

The raw data supporting the conclusions of this article will be made available by the authors, without undue reservation.
