# Supplementary material for: Screening for psychotherapy readiness with the University of Rhode Island Change Assessment Scale and the Readiness for Psychotherapy Index
Source: Front Psychol. 2025 Aug 12;16:1530713. doi: 10.3389/fpsyg.2025.1530713 (PMC12379006; doi:10.3389/fpsyg.2025.1530713)
Supplement: Supplementary file 1 [file Data_Sheet_1.pdf]

Readiness for Psychotherapy Index (RPI)

Dette spørgeskema vil hjælpe os med at forstå dig bedre.
Ud for hvert spørgsmål skal du vælge den svarmulighed der passer bedst.

Nr.		Meget uenig	Uenig	Hverken enig eller uenig	Enig	Meget enig
1	Mine problemer vil forsvinde af sig selv med tiden.					
2	Jeg har ikke den store lyst til at arbejde hårdt i terapien.					
3	Jeg vil ikke have problemer med at være fuld-stændigt ærlig og åben overfor terapeuten.					
4	Mine problemer gør mig ulykkelig					
5	Selvom det er svært for mig at arbejde med nogle ting i terapien vil jeg blive ved til den slutter.					
6	Jeg vil arbejde hårdt imellem terapitimerne med de ting jeg lærer i terapien.					
7	Jeg skammer mig over mine problemer.					
8	Det vil være for smertefuldt at fortælle min terapeut alt om mine problemer.					
9	Mine problemer gør mig meget følelsesmæs-sigt utilpas.					
10	Der er nogle ting ved mig jeg ikke ønsker min terapeut skal vide.					
11	Selvom terapien gør mig utilpas vil jeg fort-sætte.					
12	Selvom jeg har nogle problemer har jeg ikke noget behov for at løse dem nu og her.					
13	Jeg tænker ærligt talt ikke jeg kan få meget ud af terapi.					
14	Terapien vil fungere bedst hvis jeg gør en stor indsats for at samarbejde med min terapeut.					
15	Jeg vil godt kunne tale frit om mine problemer med min terapeut.					
16	Jeg har behov for at begynde at arbejde med mine problemer med det samme.					
17	Mine problemer generer mig ikke ret meget.					

Nr.		Meget uenig	Uenig	Hverken enig eller uenig	Enig	Meget enig
18	Jeg kan godt udholde ubehag i terapien fordi jeg ved det vil hjælpe mig i det lange løb.					
19	Jeg vil kunne tale med terapeuten om alt i mit privatliv.					
20	Mine problemer påvirker rigtig meget hvordan jeg har det.					

Readiness for Psychotherapy Index (RPI) er originalt udviklet af Ogrodniczuk, Joyce og Piper (2009).
